# Management Options for Advanced Low or Intermediate Grade Gastroenteropancreatic Neuroendocrine Tumors: Review of Recent Literature

**DOI:** 10.1155/2017/6424812

**Published:** 2017-05-16

**Authors:** Vladimir Neychev, Electron Kebebew

**Affiliations:** ^1^Department of Surgery, University Hospital “Alexandrovska”, Medical University, Sofia, Bulgaria; ^2^Endocrine Oncology Branch, National Cancer Institute, National Institutes of Health, Bethesda, MD, USA

## Abstract

Our understanding of the biology, genetics, and natural history of neuroendocrine tumors (NETs) of the gastrointestinal tract and pancreas has improved considerably in the last several decades and the spectrum of available therapeutic options is rapidly expanding. The management of patients with metastatic low or intermediate grade NETs has been revolutionized by the development of new treatment strategies such as molecular targeting therapies with everolimus and sunitinib, somatostatin analogs, tryptophan hydroxylase inhibitors, and peptide receptor radionuclide therapy that can be used alone or as a multimodal approach with or without surgery. To further define and clarify the utility, appropriateness, and the sequence of the growing list of available therapies for this patient population will require more high level evidence; however, data from well-designed randomized phase III clinical trials is rapidly accumulating that will further stimulate development of new management strategies. It is therefore important to thoroughly review emerging evidence and report major findings in frequent updates, which will expand our knowledge and contribute to a better understanding, characterization, and management of advanced NETs.

## 1. Introduction

Neuroendocrine tumors (NETs) of the gastrointestinal tract and pancreas are rare and heterogeneous, but clinically important group of neoplasms with unique tumor biology, natural history, and clinical management issues [1, 2]. NETs develop from the dispersed neuroendocrine cells of the gastrointestinal tract (GI) mucosa (also called “carcinoids”) and the pancreatic islet cells. Approximately 85% of NETs are sporadic and the remainder occur as part of familial cancer syndromes including multiple endocrine neoplasia-type 1 (MEN1), von Hippel-Lindau disease (VHL), von Recklinghausen's disease (neurofibromatosis 1, NF1), and tuberous sclerosis (TS) [3–5].

Neuroendocrine cells are one of the largest groups of hormone-producing cells in the body. At least 13 distinct gut neuroendocrine cells exist, all of which may develop tumors and/or oversecrete various bioactive peptides or amines including serotonin, somatostatin, histamine, and gastrin. Hypersecretion of these hormones can result in significant morbidity and mortality. Up to 20% of patients with NETs may develop carcinoid syndrome: flushing, abdominal pain, diarrhea, bronchoconstriction, and carcinoid heart disease [6].

Treatment of NETs is largely dependent on the functional status and the stage. Advanced NETs are characterized by local invasion and regional and distant metastases. While the treatment of localized NETs is surgical resection, a variety of therapeutic options are available for patients with advanced NETs. These include medical control of excess hormone levels and associated symptoms, cytoreductive surgery for patients with advanced disease, radioembolization, chemoembolization, systemic chemotherapy, interferon, long-acting somatostatin analogs, and peptide receptor-targeted radionuclide therapy. When to utilize a given option, what combination therapeutic approach should be used, how long treatment should be continued, and in what subgroup of patients should a particular treatment option be used are a work in progress.

## 2. Classification, Epidemiology, and Prognosis

The annual incidence of NETs has been increasing worldwide [1, 5, 7, 8]. Whereas early studies have reported incidence rates of <1 per 100,000 persons per year, recent age-adjusted epidemiologic studies have shown a significant, more than fivefold, increase in NETs incidence from 1973 to 2005 [5, 7, 8]. Based on data from the National Cancer Institute's Surveillance, Epidemiology and End Results (SEER) cancer registry database, the annual incidence of NETs was estimated to be 7.8 per 100,000 persons in 2013 [5]. The prevalence of NETs has been estimated as 35 per 100,000 and may be considerably higher if clinically “silent” tumors are included [9].

A population-based study conducted in Canada showed that the incidence of NETs has markedly increased over the course of 15 years from 2.48 cases per 100,000 people per year in 1994 to 5.86 per 100,000 per year in 2009 [10]. Outside USA, Canada, and Europe, epidemiological surveys have been conducted in Japan showing that the number of treated patients with NETs in 2010 increased approximately 1.2-fold compared to 2005 and the number of new incidences of NETs in 2010 was nearly 2-fold greater than in 2005 [11].

NETs are generally classified into functioning (hormone hypersecreting) or nonfunctioning (clinically “silent”) tumors, based on their ability to produce hormone-associated symptoms [12]. However, other classification systems with many common themes, such as the distinction of well-differentiated (low and intermediate grade) from poorly differentiated (high-grade) NETs and the tumor proliferative index, have been used over the past 5 decades (Table 1). In general, well-differentiated, low or intermediate grade NETs have a relatively indolent behavior with slow progression but poorly differentiated tumors may exhibit highly aggressive behavior with rapid metastatic spread that is clinically indistinguishable from pancreatic adenocarcinoma or small-cell lung cancer [3]. Fortunately, poorly differentiated tumors account for only a small subset of all NETs.

Recent large, epidemiologic studies have shown that majority of NETs (60–90%) are clinically nonfunctioning, well-differentiated, slow-growing neoplasms diagnosed, in most instances, incidentally during unrelated procedures or diagnostic tests [1, 4, 6, 13]. As a result of this insidious biological behavior, many patients with NETs have advanced disease at diagnosis, with regional or distant metastasis observed in up to 80% of patients [7, 13].

Even though there has been a significant improvement in the diagnosis and management of NETs, no significant change in survival has been observed over the last 30 years [4, 5]. Reported survival times for patients with advanced NETs in population-based and institutional series are highly variable likely due to differences in tumor biology, classification, treatment modalities, and patient selection. According to data from the SEER database, the median overall survival (OS) for patients with metastatic NETs was two years [7], whereas, in a large institutional database, the median OS for a similar group of patients was 5.8 years [14]. According to the European Neuroendocrine Tumor Society (ENETS) consensus statement, the median OS of nonfunctional NETs was 38 months with a 5-year survival rate of 43% [15].

## 3. Management of Advanced NETs

Currently available therapeutic options for patients with NETs can be summarized and subdivided into four major categories: (1) locoregional therapeutic options, including surgical resection or liver-directed therapies such as radiofrequency ablation (RFA), transcatheter arterial hepatic embolization (TAE), transcatheter arterial hepatic chemoembolization (TACE), or radioembolization therapy (TARE), (2) somatostatin receptor targeting therapeutic options, including somatostatin analogs and peptide receptor radionuclide therapy (PRRT), and tryptophan hydroxylase inhibitors, (3) molecular and mutation targeting therapy, and (4) systemic chemotherapy.

## 4. Locoregional Therapeutic Options

### 4.1. Surgical Resection

Surgical resection is the mainstay of treatment for patients with early-stage disease; however, the extent, timing, and effect of surgical intervention for advanced, metastatic NETs remain controversial. NETs most commonly metastasize to the locoregional lymph nodes and liver and 25% to 93% of patients will develop liver metastases [16]. Prospective randomized data on the treatment of liver metastases of NETs are lacking and there is considerable debate regarding the optimal surgical management [17–20]. Despite the lack of randomized control data, the current concept for the surgical treatment of patients with advanced NETs is that, when feasible, aggressive surgical resection is associated with the best symptom-free and long-term survival results [4, 16, 20, 21]. More specifically, the ENETS revised consensus statement emphasized that resection should be the first-line treatment option for patients with advanced NETs if 90% or more of the disease burden can safely be resected [22]. However, only 5% to 20% of patients with advanced NETs meet this “conventional” criterion and there is no evidence from randomized studies in the literature for the role of cytoreductive surgery in unresectable metastatic NETs [23].

### 4.2. Role of Aggressive Surgical Resection

A number of retrospective and small prospective studies, summarized previously in several elegant systematic reviews, have analyzed the potential role of surgery for advanced metastatic NETs [24–29].

Some have shown that *R*0/*R*1 resection of primary tumor and liver metastases in patients with advanced pancreatic NETs (*n* = 9) resulted in an overall survival (OS) of 71% (median: 76 months) and progression-free survival (PFS) of 5% (median: 21 months) at 5 years [31]. Others reported that resection of the primary tumors and liver metastases in 170 patients (108 functional tumors) resulted in OS of 61% and 35% at 5 and 10 years, respectively, without differences between GI and pancreatic NETs or functional and nonfunctional tumors [32]. Recurrence rate of complete (*R*0) versus incomplete (*R*2) resection was 76% and 91% at 5 years (median time to recurrence 30 months versus 16 months, resp., *p* = 0.0004). Symptoms relief was achieved in 104 of 108 patients (96%), but the recurrence rate of symptoms was 59% [32]. A retrospective study of 54 patients with advanced metastatic NETs (30% functional tumors) has shown that patients with resection of primary tumor site only (*n* = 42) had better survival than patients without surgery at all (*n* = 12) (60% versus 30% at 5 years, resp.; *p* = 0.025) [33].

The impact of aggressive surgical resection on performance status and symptoms control was evaluated in symptomatic patients (*n* = 30) with advanced GI NETs and an extensive liver involvement [34]. All patients had surgical exploration during which some patients (*n* = 22) had an adjunct RFA of one or more liver lesions. Postoperatively, 5-hydroxyindoleacetic acid (HIAA) decreased by 50% in all patients with symptomatic improvement reported in 25 patients (83%). Mean pre- and postoperative Karnofsky physical performance scores were 55 and 85, respectively (*p* < 0.02) [34].

Analysis of records from an international database of eight major hepatobiliary centers including 339 patients with metastatic NETs demonstrated that aggressive surgery resulted in OS of 74% and 51%, at 5 and 10 years, respectively; however, disease recurred in 94% of patients at 5 years. Patients with hormonally functional NETs who had *R*0/*R*1 resection benefited the most from surgery (*p* = 0.01) [35].

Assessment of the impact of surgical (*n* = 55) versus medical (*n* = 30) treatment on quality of life (QoL) of patients with advanced metastatic NETs and symptoms of hormonal overproduction showed that less than one-fourth of patients experienced a significant improvement in QoL after treatment [36]. There was no difference in the improvement in overall QoL in respect to treatment modality; however, a lower proportion of patients were dissatisfied with surgery versus nonsurgical therapy (5.4% versus 9.4%, *p* = 0.001) [36].

A retrospective study evaluated the efficacy of multimodal hepatic cytoreduction including resection, radiofrequency ablation, chemoembolization, or combined therapy in 15 symptomatic patients with advanced hepatic metastases from GI NETs. At a mean follow-up of 29 months, 6 patients (40%) had stable disease, 8 (53.3%) had progression of disease, and 1 (6.6%) had no evidence of disease. The median symptom relief period was 12 months and OS was 57 months (mean) [37].

Another study of 41 patients with an extensive hepatic resection for NETs with liver-localized metastases and median follow-up of 51 months (range: 7 to 165) showed that recurrences developed in 32 patients (78%) mainly in the liver after a median of 19 months (range: 2 to 79) [38]. Five-year OS and disease-free survival (DFS) rates were 79% and 3%, respectively [38]. Another such study investigated the effect of the resection of liver metastases in 172 patients with advanced NETs and revealed that surgery resulted in a median OS of 9.6 years (range: 89 days to 22 years) [39].

A similar analysis of records of 72 patients with curative (*n* = 39) or palliative (*n* = 32) resection of pancreatic NETs with hepatic metastases revealed an OS at 1, 5, and 10 years of 97%, 60%, and 45%, respectively [40]. Among the patients with a complete (*R*0) resection, the 1- and 5-year disease-free survival were 53.7% and 10.7%, respectively. For patients undergoing debulking of 90% tumor burden, the 1- and 5-year survival free of progression were 58.1% and 3.5%, respectively [40]. Another single-center study of 204 patients with hepatic NET metastases showed that patients with an *R*0 resection (*n* = 38) had an excellent OS of 90.4% at 10 years, while *R*1 (*n* = 23) or *R*2 (*n* = 33) resection had a 10-year survival of 53.4 and 51.4%, respectively [41]. The majority of the patients (53.9%) were not surgical candidates and had a poor 10-year survival rate of 19.4%. Partial or complete control of endocrine-related symptoms was achieved in all patients with functioning tumors following surgery [41].

A prospective study evaluated outcomes from a complete cytoreductive surgery of peritoneal metastases in 41 patients with advanced NETs with (*n* = 28) or without (*n* = 13) hyperthermic intraperitoneal chemotherapy (HIPC) [42]. Sixty-six percent of these patients also had resection of liver metastases during the same procedure. OS was 69% and 52%, and DFS was 17% and 6% at 5 and 10 years, respectively. While the OS was not impacted by the performance of HIPC, DFS was greater in the HIPC group (66%) when compared to patients without HIPC (47%), at 5-year follow-up (*p* = 0.018) [42].

A recent study reported outcomes of curative liver resection for advanced NETs in 376 patients [43]. The median and 5-year DFS were 4.5 years and 46%, respectively. The probability of being cured by liver surgery was 44% with time to cure of 5.1 years. A multivariable cure model found type and grade of NETs, as well as rate of liver involvement, to be independent predictors of cure. The cure fraction for patients with well-differentiated GI NETs or functional pancreatic NETs and liver involvement < 50% was 95%, while the presence of all the three unfavorable prognostic factors (nonfunctional pancreatic NET, liver involvement > 50%, and moderate/poor differentiation) rendered cure fraction of 8% [43]. Another recent large-cohort study of 800 patients with cytoreductive surgery for advanced NETs showed a median OS for patients with pancreatic NETs of 124 months (5-, 10-, and 20-year OS rates were 67%, 51%, and 36%, resp.) [44]. Median OS for patients with GI NETs was 161 months (5-, 10-, and 20-year OS rates were 84%, 67%, and 31%, resp.) [44].

### 4.3. Role of Liver Transplantation

The potential role of liver transplantation in management of patients with advanced NETs remains controversial and although it is not generally recommended, the available data suggest that it may be an option in highly selected patients with well-differentiated, functional NETs with extended liver metastases refractory to multiple systemic treatments and excluded extrahepatic disease by optimized staging [27, 45, 46]. Some series have reported promising outcomes in well-selected patients after liver transplantation with an OS of 70 to 73% at 5 years from diagnosis of liver metastases [47, 48]. Others have reported less favorable results, with a posttransplant 5-year survival rate as low as 49% to 58% in transplant recipients [49, 50].

In summary, despite the lack of large randomized control studies, there is a growing body of evidence from an increasing number of retrospective and prospective studies showing that surgical resection of advanced metastatic NETs with curative intent or for palliation provides favorable oncological outcomes with significant alleviation of symptoms and improvement of survival. While most studies make it clear that the aim of surgery should be *R*0 status, the role of surgical debulking (*R*2) in patients where an *R*0 resection cannot be achieved remains debatable. It has been shown by some that selected patients may benefit from *R*1 or *R*2 resection especially for symptoms control and that there may be a potential benefit from resection of the primary lesion only in patients with otherwise unresectable liver metastases [26]. On the other hand, others have shown less favorable results with no evidence to support the use of a *R*2 resection to improve either OS or QoL in patients with advanced metastatic NETs [25].

Despite exhaustive surgery hepatic or extrahepatic recurrences are frequent and may develop early [24–29]. Thus, it is becoming increasingly clear that new, perhaps combination (surgical and medical) therapeutic strategies to improve the outcomes of patients with advanced NETs need to be explored [51].

### 4.4. Minimally Invasive, Liver-Directed Therapy

Due to the insidious behavior of NETs and indolent course of the disease, many patients will present with liver metastases, which is associated with a poor prognosis [52]. In contrast to normal hepatic parenchyma that depends largely on portal venous circulation, the hypervascular liver NETs metastases rely primarily on hepatic artery blood supply. Thus, during the tumor ischemia produced by TAE liver-directed therapy, normal liver tissue will be relatively protected. Compared with systemic chemotherapy or radiation, TACE and TARE combining delivery of radio or chemotherapeutics with embolization of tumor arterial blood supply offer several advantages, including significantly increased local, peritumoral drug or radioactivity concentration and local cytotoxic effects with decreased systemic toxicities [53]. Various chemotherapeutic agents including doxorubicin, streptozocin, cisplatin, 5-fluorouracil, and mitomycin-C alone or in combination as well as beta-emitting radioactive agents such as 90Yttrium have been used for TACE and TARE; however, there is no consensus on which therapeutic agent to use, and it remains unclear if TACE and TARE offer outcome advantage over bland embolization for this patient population [52, 54]. In addition, no significant difference in safety profiles of these liver-directed modalities was found with the most common adverse events being nausea, vomiting, abdominal pain, and fever [52]. Although the number of studies investigating RFA treatment of NET liver metastases is limited, percutaneous or laparoscopic use of RFA alone or in combination with surgery has been shown to be effective in both relieving symptoms and achieving local control of NET liver metastases [22].

None of the liver-directed techniques has been shown to be more beneficial than the others and histological proof of the complete destruction of tumor foci is difficult to obtain; however, these minimally invasive therapies are showing increasingly favorable results including increased progression-free survival (PFS) and OS rates and improved symptomatic response [55].

A prospective multicenter phase II study evaluated safety and dose reproducibility of 90Yttrium TARE in the treatment of patients with diverse liver metastases, including a relatively large cohort of patients with NETs (*n* = 43) [56]. For patients with advanced NETs, disease control rate at 1 year was 93% and median PFS and medium survival were not achieved [56]. A meta-analysis of studies with 90Yttrium TARE in patients with metastatic NETs demonstrated an objective response rate (OR) of 50% and disease control rate of 86% [57].

Another recent systematic review analyzing benefits and risks of hepatic resection versus nonsurgical treatments in patients with resectable liver metastases showed no robust evidence that a liver resection was superior to any other liver-directed therapies including TAE, TACE, TARE, and RFA in improving OS, PFS, or QoL [25].

More recently, a large prospective study evaluated outcomes of hepatic resection, RFA, TACE, systemic therapy, or observation as separate or combined procedures in 649 patients with advanced NETs metastatic to the liver [44]. Median and 5- and 10-year OS for each treatment group were as follows: patients with hepatic resection (*n* = 58), 160 months, 90%, and 70%, respectively; RFA (*n* = 28), 123 months, 84%, and 55%, respectively; TACE (*n* = 130), 66 months, 55%, and 28%, respectively; systemic therapy (*n* = 316), 70 months, 58%, and 31%, respectively; and observation (*n* = 117), 38 months, 38%, and 20% [44].

## 5. Somatostatin Analogs, Tryptophan Hydroxylase Inhibitors, and Somatostatin Receptor Targeting Therapy

### 5.1. Somatostatin Analogs Therapy

A unique feature of most NETs is the expression of somatostatin receptors (SR) by the tumor cells. There are five different SR subtypes and more than 80% of NETs express multiple subtypes, with a predominance of receptor subtypes 2 and 5 [13]. Somatostatin is an endogenous SR agonist which inhibits the secretion of a broad range of hormones from the endocrine system, including serotonin, insulin, glucagon, vasoactive intestinal peptide, and gastrin [58]. Somatostatin has limited clinical use due to its short half-life (<3 min); however, somatostatin analogs (SA) such as octreotide, lanreotide, and pasireotide and their long-acting release formulations (LAR) play an important role in the treatment of patients with NETs [59–61]. With the exception of insulinoma that is less responsive to SA due to lower expression of SR and gastrinoma treated initially with proton pump inhibitors, SA are generally considered the first-line treatment for patients with functional NETs and symptoms of hormonal hypersecretion [62–64]. Although SA are mainly used to treat symptoms of hormone hypersecretion, there is emerging evidence from several ongoing or completed clinical trials suggesting that SA have antiproliferative activity (Table 2).

A phase III, randomized, placebo-controlled study (PROMID trial) demonstrated prolonged PFS (14.3 versus 6.0 months in the placebo arm) in patients with well-differentiated small bowel NETs and low hepatic tumor volume treated with octreotide LAR [65]. The most frequently observed severe adverse events, regardless of causal relationship to treatment, were observed more often in the octreotide LAR arm and included diarrhea and flatulence [65]. A follow-up PROMID study tested the effect of octreotide LAR on OS and found patients assigned to octreotide and placebo had only slightly different OS, 84.7 versus 83.7 months, respectively (*p* = 0.51) [66].

Additionally, a recent phase III, randomized, double-blind, controlled study (CLARINET trial) reported that extended-release aqueous-gel formulation of lanreotide has an antitumor effect against a broader spectrum of nonfunctional enteropancreatic NETs with significantly improved PFS (median not reached versus 18.0 months in the placebo arm) [62]. The most common treatment-related adverse event was diarrhea (26% of the patients in the lanreotide group and 9% in the placebo group) [62]. The expanding role of SA in the treatment of NETs beyond their symptom controlling properties has been further elegantly outlined in several recent reviews [63, 67, 68].

Although octreotide and lanreotide have been shown to be increasingly useful for the treatment of advanced NETs, drug resistance or escape phenomenon has been reported to possibly account for the decreased efficacy of the drugs after 6 to 18 months of therapy [13, 68]. A phase II, open-label, multicenter study of pasireotide, an SA with high affinity for SR types 1, 2, 3, and 5, showed that this drug was effective in 27% of patients with advanced NETs whose symptoms (diarrhea/flushing) were inadequately controlled by octreotide LAR [69]. The most common, mild or moderate severity drug-related adverse events were nausea (27%), abdominal pain (20%), weight loss (20%), and hyperglycemia (16%). Another phase II clinical trial evaluating the antiproliferative activity of pasireotide in treatment-naive patients with advanced NETs showed a median PFS of 11 months with the most favorable effect observed in patients with low hepatic tumor burden, normal baseline chromogranin A, and high expression of SR type 5 in tumor cells [70]. The most common adverse effect observed was hyperglycemia in 79% of patients [70].

### 5.2. Tryptophan Hydroxylase Inhibitors

Somatostatin analogs alone or in combination with surgery, radiation, or embolization have been successfully used for control of symptoms in patients with advanced NETs who develop carcinoid syndrome. Yet, a significant proportion of these patients remain refractory to treatment with SA and suffer from debilitating symptoms of serotonin overproduction [75]. Telotristat etiprate (TE) is a novel oral inhibitor of tryptophan hydroxylase, the rate-limiting enzyme for conversion of tryptophan to serotonin, that showed promising efficacy and safety profiles in several phase I and phase II trials conducted in the USA and Europe [76, 77]. The primary outcomes in the phase II clinical trials were (1) biochemical response (at least 50% reduction or normalization in urinary excretion of serotonin breakdown product HIAA) and (2) clinical response defined as a reduction in the daily mean number of bowel movement of at least 30% from baseline or normalization in the daily mean number per week (achievement of a daily mean ≤ 3 bowel movements per week). The results of these studies were consistent and showed a significant reduction in bowel movement frequency (with patient reported improvement of QoL) and a sustained, significant decrease in urinary HIAA. These early clinical studies supported the conduct of a placebo-controlled, double-blind, phase III clinical trial (TELESTAR) of 211 patients with carcinoid syndrome randomly assigned to placebo (*n* = 71) or TE 250 mg (*n* = 70) or TE 500 mg (*n* = 70), each given 3 times per day for 12 weeks [75, 78]. Patients who qualified for TELESTAR were on stable-dose SA therapy at enrolment, continued SA therapy throughout study period, and had ≥4 bowel movements per day. TELESTAR was accompanied by a satellite study (TELECAST) with the same randomization design that included patients who did not qualify for TELESTAR, because they had <4 bowel movements per day or do not tolerate or will not take SA. Median bowel movement frequency was 5.7 per day (TELESTAR) and 2.5 per day (TELECAST), and mean urinary HIAA was 84 mg/24 hours. The results of TELESTAR study showed that compared to the placebo group (*n* = 45) patients taking 250 mg (*n* = 45) and 500 mg (*n* = 45) TE experienced a median reduction of 0.81 (*p* < 0.001) and 0.69 (*p* < 0.001) bowel movements daily, respectively. There was a significant and durable reduction in absolute mean urinary HIAA from baseline of patients on 250 mg (*n* = 32) and 500 mg (*n* = 31) TE with 40.13 mg/24 hours and 57.73 mg/24 hours, respectively, compared to placebo group (*n* = 29) that showed an increase with 11.47 mg/24 hours (*p* < 0.001). Integrated safety analyses showed no serious adverse effects with slightly higher rates of nausea, constipation, and depression reported with TE.

In summary, TE has the potential to reduce serotonin levels and become a valuable alternative for the treatment of patients with advanced NETs and carcinoid syndrome refractory to treatment with SA.

### 5.3. Peptide Receptor Radiotherapy (PRRT)

The potential therapeutic benefits of nuclear medicine using radiolabeled SA are of great practical research interest and are currently being used in multiple European medical centers for the treatment of patients with NETs [79]. PRRT is based on the principle used in 111Indium-labeled octreotide scintigraphy and 68Gallium-labeled DOTA PET/CT but utilizes SA radiolabeled with *β*-emitters such as 177Lutetium-DOTATATE (177Lu-DOTATATE) and 90Yttrium-DOTATOC (90Y-DOTATOC). Such an approach has been shown as an effective treatment option in patients with advanced, metastatic NETs in multiple phase I and II clinical trials (Table 3) [64, 80–82].

One large retrospective study of more than 500 patients with advanced NETs treated with 177Lu-DOTATATE showed complete or partial response (PR) in 30% and minor response in 16% of the patients [80]. The most commonly reported acute (within 24 hours) toxicities included nausea, vomiting, and abdominal pain; subacute hematologic toxicity (4 to 8 weeks after administration) occurred in 9.5% of patients and alopecia in 62%. Nine patients experienced serious delayed toxicities, including renal insufficiency (*n* = 2), liver toxicity (*n* = 3), and myelodysplastic syndrome (*n* = 4) [80]. The therapeutic effect of 90Y-DOTATOC has been evaluated in several phase I and II clinical trials that showed modest response rates, ranging from 25% to 30% with renal toxicity being the most common dose-limiting adverse event (Table 3) [91, 92].

Although the majority of available results of PRRT benefits are from retrospective studies or phase I and II trials, the first phase III, randomized, multinational clinical trial of PRRT comparing therapeutic effect of 177Lu-DOTATATE (Lutathera®) to high dose octreotide LAR (NETTER-1 study) has been recently completed with promising findings [83, 93, 94]. Patients with inoperable, grade 1 or 2, progressive, somatostatin receptor positive midgut NETs were randomized (1 : 1) to receive Lutathera (*n* = 116) every 8 weeks (administered 4 times) versus octreotide LAR 60 mg (*n* = 113) every 4 weeks. The primary endpoint was PFS and the secondary endpoints included objective response rate (OR), OS, time to progression (TTP), safety, tolerability, and health-related QoL. At the time of statistical analysis, the median PFS was not reached for Lutathera group and was 8.4 months with 60 mg octreotide (*p* < 0.0001). Altogether, 23 confirmed disease progressions or deaths were observed in the Lutathera group versus 67 in the octreotide LAR group. The safety profile was consistent with the safety information generated in the phase I-II clinical trials reviewed above [94]. As a result of this phase III trial that provided strong evidence for a clinically meaningful benefit for patients with advanced NETs treated with Lutathera, this novel compound has received orphan drug designation from the European Medicines Agency (EMA) and the US Food and Drug Administration (FDA).

## 6. Molecular and Mutation Targeted Therapy

Our knowledge of the genetic alterations present in sporadic and familial NETs has improved significantly. Mutations in driver oncogene and tumor suppressor genes have been identified in most NETs [71, 72, 95]. Overexpression of growth factors and their receptor such as vascular endothelial growth factor* (VEGF)*,* VEGF* receptor* (VEGFR)*, platelet-derived growth factor* (PDGF)*, and* PDGF* receptor* (PDGFR)* [96] and/or somatic mutations in* MEN1*,* DAX*,* ATRX*, and* TP53* and the genes of mammalian target of rapamycin* (mTOR)* signaling pathway are common in NETs [7, 97].

### 6.1. Everolimus: Targeting Altered* mTOR* Pathway

Several studies, using whole-exome-sequencing approach and expression profiling, have consistently identified somatic mutations in and or activation of the* PI3K*/*AKT*/*mTOR* pathway as a common event in NETs [96, 97]. The* mTOR* pathway has a central role in cancer cell growth, proliferation, differentiation, and apoptosis. Tuberous sclerosis 2* (TSC2)*, phosphatase and tensin homolog* (PTEN)*,* PIK3CA*, and fibroblast growth factor 13* (FGF13)* are among the key modulators of the* mTOR* pathway [98, 99]. Several studies of global gene expression profiling in a large panel of pancreatic NETs showed that* TSC2* gene and* PTEN* gene were downregulated in most tumors, and their low expression was significantly associated with shorter disease-free survival and OS [100]. In addition, high* FGF13 *expression level was significantly associated with liver metastasis and shorter disease-free survival [100]. A recent whole-exome sequencing study also revealed the presence of somatic mutations in* MEN1, DAXX, ATRX, TSC2, PTEN*, and* PIK3CA* genes in the majority of sporadic NETs [97]. Furthermore, patients with NETs harboring these somatic mutations had a longer survival when compared to patients with wild type* MEN1* and/or* DAXX/ATRX* [97].

Activation of the* mTOR* pathway has also been implicated in several familial cancer syndromes (tuberous sclerosis (TS), neurofibromatosis type 1 (NF1), and Hippel-Lindau diseases (VHL)) associated with the development of NETs [101, 102]. Findings of these and other studies further support the critical role of the* mTOR* pathway in NETs and add to the wealth of data that has spurred the clinical development of* mTOR* inhibitors as a treatment option for patients with advanced NETs (Table 2).

Everolimus is a well-studied oral* mTOR* inhibitor. A phase II trial of everolimus in combination with long-acting octreotide in patients with advanced low to intermediate grade NETs (30 carcinoid and 30 islet cell tumors) demonstrated a partial response rate of 20% and median PFS of 15 months [103, 104]. A follow-up phase II trial, which randomized patients with metastatic pancreatic NETs (who experienced progression on or after chemotherapy) to everolimus or everolimus in combination with long-acting octreotide, showed a significantly longer median PFS in patients receiving combination therapy as compared to everolimus alone (16.7 months versus 9.7 months) [104]. More recently, a multicenter double-blind placebo-controlled phase III trial (RADIANT-3) comparing everolimus to placebo in 410 patients with progressive, advanced low or intermediate grade NETs showed that median PFS was improved with everolimus therapy (11.0 months versus 4.6 months) [72]. The most common adverse events in the everolimus group versus placebo group were stomatitis (64% versus 17%), rash (49% versus 10%), diarrhea (34% versus 10%), fatigue (31% versus 14%), and infections (23% versus 6%). The results of these studies have led to the approval of everolimus by the European Medicines Agency in the European Union and by the Food and Drug Administration in the USA for the treatment of progressive, unresectable, locally advanced or metastatic, low or intermediate grade pancreatic NETs.

In the follow-up, randomized, double-blind, placebo-controlled, phase III RADIANT-4 trial, 302 patients with advanced, progressive, well-differentiated, nonfunctional NETs were randomized to everolimus 10 mg per day orally or placebo, both with supportive care [74]. The primary endpoint was PSF, and OS and quality of life were secondary endpoints. Median PFS of 11.0 months was significantly improved in the everolimus group compared to 3.9 months in the placebo group (*p* < 0.00001). Although not statistically significant, the results of the first preplanned interim OS analysis indicated that everolimus might be associated with a reduction in the risk of death [74].

### 6.2. Sunitinib: Targeting Key Drivers of Angiogenesis

The highly vascular nature of NETs led to initial interest in investigating neoangiogenesis in NETs. A number of studies have found elevated expression of several cellular growth factors and their receptors in NETs:* VEGF*,* VEGF* receptor* (VEGFR)*, platelet-derived growth factor* (PDGF)*,* PDGF* receptor* (PDGFR)*, stem cell factor receptor* (c-KIT)*, and epidermal growth factor receptor (EGFR) [96]. Many of these receptors with their respective growth factor ligands function as tyrosine kinases (RTKs) directly and indirectly regulating tumor growth, survival, and angiogenesis. The hypothesis that inhibiting these targets in concert will result in broad antitumor efficacy in patients with NETs has been studied in several clinical trials (Table 2).

Sunitinib malate is a small molecule kinase inhibitor with activity against a number of tyrosine kinase receptors, including* VEGFR*,* PDGFR*,* KIT*,* RET*, and FMS-like tyrosine kinase-3* (FLT3)* [105]. A phase II trial in patients with advanced NETs who received sunitinib demonstrated a median time to tumor progression of 7.7 and 10.2 months in patients with pancreatic NETs and carcinoid tumors, respectively [96]. A follow-up randomized, double-blind phase III trial comparing the response of 86 randomly selected patients given sunitinib with that of 85 patients on placebo demonstrated significant improvement in median PFS of patients on sunitinib (11.4 months versus 5.5 months) [71]. Moreover, patients treated with sunitinib showed early signs of an increase in overall survival. The most common adverse events associated with sunitinib treatment were diarrhea, nausea, asthenia, vomiting, and fatigue; each occurred in 30% or more of patients [71]. Based on these findings, sunitinib was approved by the European Medicines Agency and by the Food and Drug Administration for the treatment of progressive, unresectable, locally advanced or metastatic pancreatic NETs.

None of the clinical trials with everolimus and sunitinib for treatment of patients with advanced NETs assessed whether response to therapy was related to the altered intracellular signaling pathways or tumor genotype. An open-label, prospective, phase II clinical trial for treatment of low or intermediate grade advanced, progressive NETs with everolimus or sunitinib based on the genetic alterations present in the tumor (NCT02315625) is now underway [51].

### 6.3. Other Molecular Therapeutics Investigated for Treatment of Advanced NETs

In addition to the approval of sunitinib and everolimus, a number of other agents targeting different RTKs, growth factors, and* mTOR* signaling pathway have been investigated as an alternative therapy for patients with advanced NETs.

In a phase II clinical trial of temsirolimus, an* mTOR* inhibitor, in 36 patients with advanced NETs, a modest intent-to-treat response rate of 5.6%, tumor control (stable disease (SD) plus PR) rate of 63.9%, median time to progression of 6 months, and 1-year OS rate of 71.5%, was observed. The most frequent drug-related adverse events of all grades included fatigue (78%), hyperglycemia (69%), and rash (64%) [106].

The activity of sorafenib, an orally active multityrosine kinase inhibitor targeting VEGFR-2, VEGFR-3, PDGFR-*β*, FLT3, c-KIT, RET B-RAF, and C-RAF, has been investigated in another phase II clinical trial in patients with gastrointestinal (*n* = 41) and pancreatic (*n* = 41) NETs [107]. It has been demonstrated that combined minor response and PR rates of gastrointestinal and pancreatic NETs were 17% and 32%, respectively, with a PFS of 6 months in 60.8% of patients with pancreatic NETs [107]. Most common grade 3-4 adverse events experienced by 43% of patients included skin reactions (20%), fatigue (9%), and gastrointestinal tract symptoms (7%) [107].

Another multikinase inhibitor, pazopanib, targeting VEGFR-1, VEGFR-2, VEGFR-3, PDGFR, and c-KIT, was studied in a phase II clinical trial in 37 patients with metastatic NETs and an overall response rate (ORR) of 24.3% and a disease control rate (complete response (CR) + PR + SD) of 75.7% was observed [108]. The most common grades 3 and 4 adverse events were proteinuria (11%), neutropenia (8%), hypertension (5%), diarrhea (5%), anorexia (5%), abdominal pain (5%), and elevation in liver transaminases (5%) [108]. An EGFR inhibitor, gefitinib, has been investigated in a phase II clinical trial in patients with advanced NETs and showed PFS of 6 months in 23 of 38 (61%) patients with gastrointestinal NETs and 9 of 29 (31%) patients with pancreatic NETs [109]. Grade 3 or 4 toxicities were infrequent and included fatigue (6%), diarrhea (5%), and rash (3%).

While the above clinical trials showed moderate clinical activity of monotherapies with sorafenib, pazopanib, gefitinib, and temsirolimus, combination therapy with somatostatin analogs and/or selective monoclonal antibodies targeting different growth factors have shown more promising results.

A recent multicenter phase II clinical trial of combination therapy with temsirolimus and bevacizumab in patients with progressive well- or moderately differentiated NETs (*n* = 56) showed very promising results with median PFS of 13.2 months and median OS of 34 months [110]. The most common grade 3 to 4 adverse events attributed to therapy were hypertension (21%), fatigue (16%), lymphopenia (14%), and hyperglycemia (14%) [110].

A prospective, multi-institutional phase II study of pazopanib and depot octreotide in patients with advanced low grade NETs (*n* = 51) demonstrated a median PFS of 12.7 and 11.7 months for gastrointestinal NETs and pancreatic NETs, respectively [111]. Significant, grade 3 or 4 toxicities were rare and included hypertension (*n* = 6), neutropenia (*n* = 3), fatigue (*n* = 3), diarrhea (*n* = 3), transaminitis (*n* = 3), hypertriglyceridemia (*n* = 2), anemia (*n* = 1), nausea (*n* = 1), pain (*n* = 1), rash (*n* = 1), syncope (*n* = 1), and confusion (*n* = 1).

Combination of depot octreotide, bevacizumab, and pertuzumab, a monoclonal antibody inhibiting the dimerization of HER2 with other HER receptors, was evaluated in a phase II clinical trial in patients with advanced well-differentiated NETs and demonstrated an ORR of 16% and a median PFS of 8.2 months [112].

## 7. Systemic Chemotherapy

Despite the demonstration of improved PFS in patients with advanced NETs treated with routinely used first-line therapeutic agents including octreotide, lanreotide, and everolimus or sunitinib, the overall tumor response rates with these agents have been less than satisfactory (Table 2) and for patients with bulky, rapidly progressive disease, and poorly differentiated NETs, these treatments are not likely to yield meaningful responses [64].

Multiple reports and phase I and II clinical trials of a variety of cytotoxic chemotherapy regimens have yielded promising results for the treatment of patients with high burden, rapidly progressing NETs. The effects of chemotherapies with alkylating agents, such as streptozocin, dacarbazine, and temozolomide, alone or in combination with the antimetabolites 5-FU, capecitabine, or the anthracycline drugs doxorubicin and epirubicin, have been evaluated, and encouraging results of ORR rates of up to 70% and PFS of more than 26 months have been reported for some but not all regimens [113–116]. A recent phase II clinical trial investigating the effect of chemotherapy with 5-FU and streptozocin combined with anti-VEGF monoclonal antibody bevacizumab in patients with advanced, progressive, well-differentiated NETs (*n* = 34) showed very encouraging results with a median PFS of 23.7 months [117].

Solid evidence from phase III randomized controlled trial is currently lacking and there is no consensus on which patient population should be treated and what treatment regimen should be chosen, and the optimal timing of the treatment remains to be established [115].

## 8. Discussion and Conclusion

Our understanding of the biology and natural history of NETs has improved considerably in the last several decades and the spectrum of available therapeutic options is rapidly expanding. The management of patients with metastatic NETs has been revolutionized by the development of new systemic and biological treatment strategies such as molecular targeted therapies with everolimus and sunitinib, PRRT, as well as revealing the antiproliferative properties of SA. The growing list of effective therapeutics with favorable toxicity profiles has given rise to novel multidisciplinary approaches in the management of patients with advanced NETs including the emerging concept of sequential treatment [118]. Growing body of evidence from randomized clinical trials comparing the effects of different therapeutic modalities makes it unclear when to apply a given option, what combination therapeutic approach should be used and in what sequence, how long treatment should be continued, and in what subgroup of patients a particular treatment option should be used.

While it is clear that a clinical trial of every possible combination therapy sequence would be practically impossible to design and conduct, several consensus statements by experts from the USA, Canada, and Europe have been recently developed for the management of patients with advanced low or intermediate grade NETs [27, 55, 64, 119–123].

Given the heterogeneity and intricate biology of NETs, as well as the complexity of management of every individual case, a general approach of treatment individualization by a dedicated multidisciplinary team is currently gaining increasing acceptance among physicians. This includes a thorough review of all patient-related and disease-related factors for every individual case such as consideration of disease extent, location, and progression, as well as tumor grade, patent's symptoms, comorbidities, and performance status. Careful consideration and, when it is needed, reevaluation of the potential benefits of specific therapeutic and supportive options at each clinical decision point along the disease course are essential to ensure most favorable outcomes [27, 120, 124].

More high level evidence will be needed to further define and clarify the utility, appropriateness, and the sequence of the growing list of available therapies for this patient population; however, data from well-designed randomized phase III clinical trials is rapidly accumulating that will stimulate further investigations into potential development of new management strategies. It is therefore important to thoroughly review emerging evidence from recent clinical trials and report major findings in frequent updates, which will expand our knowledge and contribute to a better understanding, characterization, and management of advanced NETs and, ultimately, help patients and their families.

## Figures and Tables

**Table 1:** Nomenclature and classification of neuroendocrine tumors.

Differentiation and grade	Mitotic count (/10 HPF)^a^	Ki-67 index (%)^b^	Traditional classification	ENETS/WHO classification	Moran et al. [30]
Well differentiated					
Low grade (grade 1)	<2	≤2	Carcinoid, islet cell, PNET	Neuroendocrine tumor, grade 1	Neuroendocrine carcinoma, grade 1
Intermediate grade (grade 2)	2–20	3–20	Carcinoid, atypical carcinoid^c^, islet cell, PNET	Neuroendocrine tumor, grade 2	Neuroendocrine carcinoma, grade 2
Poorly differentiated					
High grade (grade 3)	>20	>20	Small-cell carcinoma	Neuroendocrine carcinoma, grade 3, small cell	Neuroendocrine carcinoma, grade 3, small cell
			Large-cell neuroendocrine carcinoma	Neuroendocrine carcinoma, grade 3, large cell	Neuroendocrine carcinoma, grade 3, large cell

HPF, high-power field; ENETS, European Neuroendocrine Tumor Society; PNET, pancreatic neuroendocrine tumor; ^a^HPF = 2 mm^2^; at least 40 fields (at ×40 magnification) were evaluated in areas of highest mitotic density. Cutoff values were taken from American Joint Committee on Cancer staging system (seventh edition); ^b^Ki67/MIB1 antibody; percentage of 2,000 tumor cells in areas of highest nuclear labeling. Cutoff values were taken from American Joint Committee on Cancer staging system (seventh edition); ^c^The term atypical carcinoid only applies to intermediate grade neuroendocrine tumor of the lung.

**Table 2:** Phase III trials of somatostatin analogs and molecular targeting therapy in advanced NETs.

Tumor type and treatment regimen	Patients (number)	ORR (%)	Median PFS (months)	Median TTP (months)	Criteria
*Pancreatic NETs*					
Raymond et al. [71]					RECIST
Sunitinib	86	9		11.4	
Placebo	8	0		5.5	
Yao et al. [72] (RADIANT-3 study)					RECIST
Everolimus	207		11.0		
Placebo	203		4.6		
*Small bowel NETs*					
Rinke et al. [65] (PROMID study)					WHO
Octreotide LAR	42	2		14.3	
Placebo	43	2		6.0	
Pavel et al. [73] (RADIANT-2 study)					RECIST
Everolimus + octreotide LAR	211		16.4		
Placebo + octreotide LAR	204		11.3		
*Small bowel and pancreatic NETs*					
Caplin et al. [62] (CLARINET study)					RECIST
Lanreotide autogel	101		Not achieved		
Placebo	103		18.0		
*Small bowel and lung NETs*					
Yao et al. [74] (RADIANT-4 study)					RECIST
Everolimus	205		11.0		
Placebo	97		3.9		

ORR, overall response rate; PFS, progression-free survival; TTP, time to progression; RECIST, Response Evaluation Criteria in Solid Tumors; LAR, long-acting release; WHO, World Health Organization tumor response criteria.

**Table 3:** Clinical trials of 177Lu-DOTATATE and 90Y-DOTATOC in advanced NETs.

Treatment regimen	Patients (number)	ORR (%)	Median PFS (months)	Criteria	Reference
Phase III studies					
NETTER-1 study					
177Lu-DOTATATE	116		Not achieved	RECIST	[83]
Octreotide LAR	113		8.4		
Phase I and II studies					
177Lu-DOTATATE	310	29	33	RECIST	[80]
177Lu-DOTATATE	26	38		RECIST	[84]
177Lu-DOTATATE	12	17		RECIST	[85]
177Lu-DOTATATE	42	32	36	RECIST	[86]
90Y-DOTATOC	58	23	17	RECIST	[87]
90Y-DOTATOC	53	23	29	RECIST	[88]
90Y-DOTATOC	90	4	16	RECIST	[89]
90Y-DOTATOC	58	9	29	RECIST	[90]
90Y-DOTATOC	21	29		RECIST	[91]

ORR, overall response rate; PFS, progression-free survival; RECIST, Response Evaluation Criteria in Solid Tumors; LAR, long-acting release.
